# Multi-dimensional Treatment Foster Care in England: differential effects by level of initial antisocial behaviour

**DOI:** 10.1007/s00787-015-0799-9

**Published:** 2015-12-10

**Authors:** Ian Sinclair, Elizabeth Parry, Nina Biehal, John Fresen, Catherine Kay, Stephen Scott, Jonathan Green

**Affiliations:** 1 Social Policy Research Unit, University of York, York, YO10 5DD England, UK; 2 University of Exeter, Exeter, UK; 3 University of Oxford, Oxford, UK; 4 University of Manchester, Manchester, UK; 5 Institute of Psychiatry, London, UK

**Keywords:** MTFC, TFCO-A, Antisocial behaviour, Conduct disorder, Treatment interaction, Controlled trial

## Abstract

Multi-dimensional Treatment Foster Care (MTFC), recently renamed Treatment Foster Care Oregon for Adolescents (TFCO-A) is an internationally recognised intervention for troubled young people in public care. This paper seeks to explain conflicting results with MTFC by testing the hypotheses that it benefits antisocial young people more than others and does so through its effects on their behaviour. Hard-to-manage young people in English foster or residential homes were assessed at entry to a randomised and case-controlled trial of MTFC (*n* = 88) and usual care (TAU) (*n* = 83). Primary outcome was the Children’s Global Assessment Scale (CGAS) at 12 months analysed according to high (*n* = 112) or low (*n* = 59) baseline level of antisocial behaviour on the Health of the Nation Outcome Scales for Children and Adolescents. After adjusting for covariates, there was no overall treatment effect on CGAS. However, the High Antisocial Group receiving MTFC gained more on the CGAS than the Low group (mean improvement 9.36 points vs. 5.33 points). This difference remained significant (*p* < 0.05) after adjusting for propensity and covariates and was statistically explained by the reduced antisocial behaviour ratings in MTFC. These analyses support the use of MTFC for youth in public care but only for those with higher levels of antisocial behaviour. Further work is needed on whether such benefits persist, and on possible negative effects of this treatment for those with low antisocial behaviour.

Trial Registry Name: ISRCTN

Registry identification number: ISRCTN 68038570

Registry URL:www.isrctn.com

## Introduction

Multidimensional Treatment Foster Care (MTFC) is an evidence-based, time-limited intervention which aims to improve the behaviour of antisocial children in out-of-home care. The model was developed by Chamberlain and her colleagues at the Oregon Social Learning Centre (OSLC), Recently subsumed under the name ‘Treatment Foster Care Oregon’ (TFCO) it draws strongly on applications of social learning theory [1]. These include parent and social skills training, contingency management, token economies, and “wraparound” programmes which target the school and follow-on placement as well as the child. Meta-analyses show that such behavioural approaches reduce antisocial behaviour among children living with their families [2]. It is less certain whether they work with hard-to-manage youth in public care, and whether their effectiveness varies with subgroups of them. This paper uses a large UK trial of MTFC to examine these questions.

The main evidence on the effectiveness of MTFC with adolescents (now called TFCO-A) comes from two randomised control trials (RCTs) in the USA which compared MTFC for young offenders with group residential care. These reported positive effects on male and female offending and days in custody and a variety of secondary outcomes [3, 4]. The latter included depression or psychotic symptoms, which are not obvious markers of antisocial behaviour [5, 6].

A Cochrane review confirmed the findings on offending and days in custody, but expressed concern about how widely the findings might apply; all the studies reviewed were based in the USA, involved the programme developers and focused primarily on offenders or custodial settings [7]. Since then two Swedish RCTs involving young people with conduct disorders have also found a trend towards positive, but not always significant or persisting, benefits from MTFC on clinical functioning and various psychological tests [8, 9].

The study of MTFC reported here used an RCT combined with a case control design to evaluate a national implementation of MTFC in England. This involved hard-to-manage young people in the public care system. Many, but not all, showed a high degree of antisocial behaviour at baseline and the sample exhibited a wide range of social and emotional impairments including PTSD and suicidal behaviour [10]. The original study had two main aims: first to determine whether the intervention improved outcomes, second to understand why this was.

This second aim is the focus of this article. We want to understand how, for whom, and in what conditions MTFC works. This should enable better targeting and thus increase effectiveness. Our findings highlight the need for this since in contrast to the American trials of MTFC and to related work with birth families, we found no significant difference in the outcome of MTFC and TAU on our primary measure of outcome [10]. Two considerations may help to explain why this was so.

First, there is evidence that the effects of MTFC and its modifications for younger children are most marked with antisocial young people. Thus, it benefits the most delinquent in the case of avoiding pregnancy [11], the worst behaved in the case of behaviour [12], and those with the most previous placements in the case of stability [13].

In keeping with this evidence the RCTs referenced above focussed on groups who were mainly living at home and further defined by offending, conduct disorders or antisocial behaviour. By contrast our sample comprised young people in out-of homecare, whose placements were at high risk of disruption and nearly half (46 %) had no criminal convictions or cautions. The average externalising score on the Child Behaviour Check List (CBCL) of those receiving MTFC in our study was 27.04 (*n* = 28, sd = 11.89) as against 36.2 (*n* = 20, sd = 12.5) in the first Swedish study. The inclusion of some less antisocial young people in our study could therefore help explain the overall negative findings compared to other MTFC studies; a possibility raised but not explored in our previous article [10].

Second, there is as yet no clear evidence that MTFC benefits young people who are not displaying antisocial behaviour. Its reported effects on other aspects of adjustment could be the direct effect of MTFC’s therapeutic support and social training and thus apply to all those with adjustment problems. However, they could also be the indirect effect of reductions in difficult behaviour leading to improved relationships with carers, and hence to other improvements. And in this case only young people entering the programme with antisocial behaviour would benefit. Thus, our analysis of overall changes may have masked improvements that did occur but only in a sub-sample of those receiving MTFC and through changes in antisocial behaviour.

Against this background and in keeping with our aim of understanding why effects occur we now test three a priori hypotheses put forward before any data had been analysed: (1) the more highly antisocial adolescents will improve more with MTFC than TAU. (2) This trend will be less apparent or even reversed among the less antisocial. (3) Improvements in the overall outcomes for some adolescents receiving MTFC will be at least partly accounted for by improvements in antisocial behaviour.

## Method

### Samples

Twenty-three English local authorities participated in an RCT combined with a case control study. Inclusion criteria were: (1) aged 10–16 years and (2) assessed by the authorities as showing complex or significant emotional difficulties and/or challenging behaviour and (3) either currently looked after but in a placement which was unstable, at risk of breakdown or not meeting their assessed needs or at imminent risk of becoming looked after long-term or at risk of custody or secure care. Exclusion criteria were severe intellectual difficulties, evidence of psychotic illness or absence of informed consent.

Of the 523 adolescents referred to the study, 56 were ineligible, 191 could not be contacted for consent, 57 refused consent and seven were not followed for a year; leaving 212 (179 observational sample and 33 RCT). Following the pre-specified protocol we combined these two samples for explanatory analyses. As discussed in more detail below the observational and RCT samples did not differ significantly on any of the key variables. There were, however, differences between those receiving and not receiving MTFC in the observational sample and these had implications for our analysis,

### Interventions

#### Multidimensional Treatment Foster Care (MTFC)

MTFC aims to reduce antisocial behaviour and promote healthy peer and adult relationships and social skills [1]. A key element is the ‘Points and Levels’ system. The young people start at Level 1 and are expected to stay on it for 3 weeks. At this level, they are supervised at all times and expected to settle with their foster family and break off contact with undesirable peers. Higher levels bring more privileges and freedom and children move between levels by earning or losing points based on a standardised daily report. In the English implementation of MTFC, young people in out-of-home care were expected to reach the highest level after 9–12 months and then move to a new placement.

In this implementation specially trained foster carers were highly supervised by a multi-disciplinary team with daily telephone contact, weekly group meetings and 24 h emergency support. The individualised treatment programme for the one child in each placement was regularly reviewed and could include therapy, skills training and education support. Work with birth family (if appropriate) or follow-on carers sought to provide a consistent approach in the next placement.

#### Treatment fidelity

Fidelity was enhanced by OSLC approval of the operating procedures of all participating agencies. For the first few years of the pilot programme all clinical team staff and foster carers were trained in the core principles, theory and practice of the MTFC model by OSLC staff who came over from the USA. OSLC also trained the national team responsible for the development, which in turn provided training, support and consultancy to the local teams and later took over the training of replacement staff and carers. Site consultants employed by the national team attended the weekly clinical meetings of local teams to ensure adherence to the model, which was almost certainly more strictly followed than would be the case in normal and less supervised practice.

We constructed a placement compliance score based on ratings by the local teams. These ratings covered the use of the daily report of behaviour, and the points and levels system, along with other key aspects of the model, including the monitoring of the young person’s daily activities, the reinforcement of desired activities, and the consistency of response to the young person’s attitudes and behaviour. Fifty-three per cent of those rated scored a maximum 32 and 80 % thirty or over.

#### Treatment as usual (TAU)

TAU was residential care (64 %), ordinary foster care (31 %) and ‘other’, mainly relatives (3 %).

### Measures

Data was collected at baseline (t1) and at 12 months (t2). Baseline data on the young people came from their social workers, their carers prior to index placement, the MTFC team, the young people themselves and reports and records available in their case files. Key data included offending, school adjustment, total problem scores for the CBCL [14] and Strengths and Difficulties Questionnaire (SDQ) [15], measures of well-being and behavioural problems, and self-reported measures from the young person.

The Health of the Nation Outcome Scales for Children and Adolescents (HoNOSCA) [16] was used to make standardised researcher ratings of social and emotional functioning at t1 and t2. Thirteen sub-scales are rated on a 5 point scale from 0 (no problems relevant to that scale) to four (severe problems which affect functioning) and in a strict order so that information used in making earlier ratings (e.g., of disruptiveness) is not then used to make later ones (e.g., poor school attendance). Total scores as well as subscale scores show good inter-rater reliability and external validity [18–20].

To utilise all available data, we combined information from all data sources, i.e., carer, social worker, young person and official reports, in a single transcription for each subject at each time point. The transcriptions were structured around the sub-scales of the HoNOSCA so that information relating to each scale was extracted from all available informant data. The information was then rated using the standard scoring system by the original transcriber and a second rater who was blind to the subject’s identity and treatment allocation.

Finally, the same data and transcripts were used by each rater (transcriber and blind rater) to produce the Childhood Global Assessment Scale (CGAS) [17], a single measure of global functional adaptation, representing functioning at home, school, with friends and during leisure time on a scale of 1 (very poor) to 100 (excellent). The measure has been widely used within child mental health settings and epidemiology and intervention studies and has been found to have high inter-rater reliability in research settings at 0.83 or 0.91 [17, 21].

The analysis used the blinded HoNOSCA and GGAS ratings unless there was a discrepancy of more than 10 points on a CGAS rating, when a third rater (also blinded) was used to review all the information and the HoNOSCA ratings. And the median rating taken (see Green et al. [10] for further details of coding procedure). The ICC for the HoNOSCA domain scores used in the analysis varied from 0.53 to 0.89 at t1 and 0.51–0.89 at t2 with 19 of the 26 ratings being at 0.7 or above. The GCAS ratings were guided by representative vignettes for each decile and showed a high level of agreement (ICC = 0.75 at t1 and 0.81 at t2).

Being based on the same information the HoNOSCA and CGAS ratings were closely related with 9 of the HoNOSCA domains accounting together for three quarters of the variance in the t1 CGAS. The measures were not, however, identical. The HoNOSCA ratings both inform the overall rating of the CGAS and indicate particular aspects of functioning. As a global rating the CGAS allowed an assessment of the significance of the different HoNOSCA dimensions in the young person’s life.

### Analysis

#### Sample trimming

The randomized and observational sub-samples did not differ significantly on any of the variables used in this article. There were, however, significant differences between those who did and did not receive MTFC in the observational sample, with the latter being significantly younger and scoring significantly lower (worse) on the CGAS at both t1 and t2.

To reduce this problem all analyses used a trimmed sample adjusted for significant baseline imbalances between the arms of the observational component of the study by using Propensity scores (see Rubin [22]). These were created through logistic regression with receipt of MTFC as dependent variable and baseline age, sex, previous placement, CGAS, and mean HoNOSCA ratings as independent ones. The independent variables were selected on the grounds that they were either logically related to the outcome measure (CGAS), or had been found in preliminary analysis to distinguish significantly between those receiving or not receiving MTFC. The other baseline variables used in this article did not contribute significantly to this predictor but as described later were used in propensity weighting within the trimmed sample. Children with propensity scores for receiving MTFC lower than any found in the MTFC sub-sample or higher than any found in the TAU sub-sample were excluded from analysis leaving a trimmed sample of 171 (MTFC = 88 and TAU-83).

#### Primary outcome

CGAS global measure at t2 adjusted for t1 CGAS was the primary outcome. We also assessed the degree to which change in CGAS at t2 reflected changes in antisocial behaviour or other dimensions of functioning by using the HoNOSCA antisocial score and a composite ‘Other problems’ score comprising the individual’s mean rating on the remaining 12 dimensions of the HoNOSCA.

#### Covariates

Four covariates were identified through a series of backwards regressions. All variables thought likely to predict outcome (t2 CGAS scores) were entered successively and only those with significant coefficients retained. The resulting ‘best predictors’ were the t1 CGAS, two measures based on whether there was evidence from the adolescent’s records that they were ever convicted or offended, or ever treated for, or diagnosed as having, a mental health problem and ‘risk,’ a composite measure designed to capture the main negative features in the baseline information. The risk score was a factor score accounting for just over a third of the variance in a component analysis of six baseline variables: the total CBCL and total SDQ scores, combined average social worker and carer ratings of well-being, a count of 13 possible difficulties noted by social workers, a measure of the young person’s self-assessed well-being and a measure of challenging behaviour based on school attendance and offending as assessed from records. It was an empirical measure which correlated 0.92 with the total CBCL T score and whose justification was that it outperformed other variables as a predictor of our main outcome (CGAS) without being derived from it.

#### Baseline antisocial status

Antisocial group status was defined using baseline HoNOSCA scale 1 ‘problems with disruptive, antisocial or aggressive behaviour’. This scale shows high inter-rater reliability in our study (ICC = 0.78 at t1 and 0.85 at t2) and internationally (ICC ≥ 0.88) [19, 20] and external validity, with correlations of 0.62 with the externalising scores of parent rated CBCL and significantly higher concurrent ratings in children independently diagnosed as having conduct disorders [18]. Participants rated as moderate (3) or severely (4) antisocial on this scale formed the ‘*high antisocial group’* (*n* = 112) and the remainder (scoring ≥2) the ‘*less antisocial group’* (*n* = 59).

#### Statistical analysis

Analyses used SPSS v 21 and involved a mix of bivariate comparisons, regression methods, propensity weighting, doubly robust estimation and mediation analysis.

### Ethics

Approval was obtained from University of York Research Ethics committee and approval from the UK Association of Directors of Social Services.

## Results

### Did the more highly antisocial adolescents improve more with MTFC than with TAU and was this trend less apparent or even reversed among the less antisocial?

Table 1 gives mean scores on our main outcome variable (the CGAS) at t1 and t2, according to antisocial group and treatment received. As can be seen, at t1 the average CGAS scores of the antisocial groups receiving MTFC or TAU were almost identical. However, between t1 and t2 the average CGAS score of the antisocial group receiving MTFC improved by an average of 9.36 points (*t* = 6.93, *df* = 66, *p* < 0.001) as against an improvement of 5.34 (*t* = 4.307, *df* = 44, *p* < 0.001) under TAU. By contrast the mean CGAS of the less antisocial group fell 0.81 (*t* = −0.474, *df* = 20, *p* = 0.64) if they received MTFC but improved by 4 (*t* = 2.387, *df* = 37, *p* = 0.022) if they received TAU.

**Table 1:** Outcome variables at baseline and follow-up by antisocial group and allocation to MTFC

Treatment group	Antisocial group		Less antisocial group		Total	
	MTFC	TAU	MTFC	TAU	MTFC	TAU
*N*	67	45	21	38	88	83
CGAS t1						
Mean	45.28	44.82^b^	50.71	53.95^b^	46.58	49.00^*b^
SD	6.82	6.29	6.54	8.38	7.11	8.59
CGAS t2						
Mean	54.64	50.16*^b^	49.90	57.95	53.51	53.72
SD	10.25	9.39	6.80	10.70	9.72	10.69
Change (t2 = t1)	9.36***^a^	5.33***^a^	−0.81^a^	4.00*^a^	6.93***^a^	4.72***^a^

* ≤ 0.05, ** ≤ 0.01 *** ≤ 0.001

^a^ *t* test paired samples

^b^ *t* test independent samples

TAU thus appeared to improve the CGAS scores of the antisocial and less antisocial groups by equal amounts. In sharp contrast MTFC appeared to benefit the antisocial group considerably but showed no, or negative, impact on the less antisocial. Consistent with this, in MTFC the antisocial group scored on average roughly five points less (i.e. ‘worse’) on the CGAS than the less antisocial at t1 (*t* = −0.3.22, *df* = 86, *p* = 0.002) but five points more (i.e. ‘better’) at t2 (*t* = 1.98, *df* = 86, *p* = 0.051).

These differences in the direction and size of change had other important consequences. At t1 MTFC and TAU did not differ significantly on our outcome variables in either the high antisocial or the less antisocial group. By t2, however, the high antisocial group had significantly higher CGAS scores if they received MTFC (*t* = 2.348, *df* = 110, *p* = 0.021) but the less antisocial group had significantly higher ones if they received TAU (*t* = 3.109, *df* = 57, *p* = 0.003). The net effect of these contrasting trends is that the average CGAS at t2 is virtually the same in MTFC and TAU (*t* = 0.136, *df* = 169, *p* = 0.892).

**Fig. 1:**
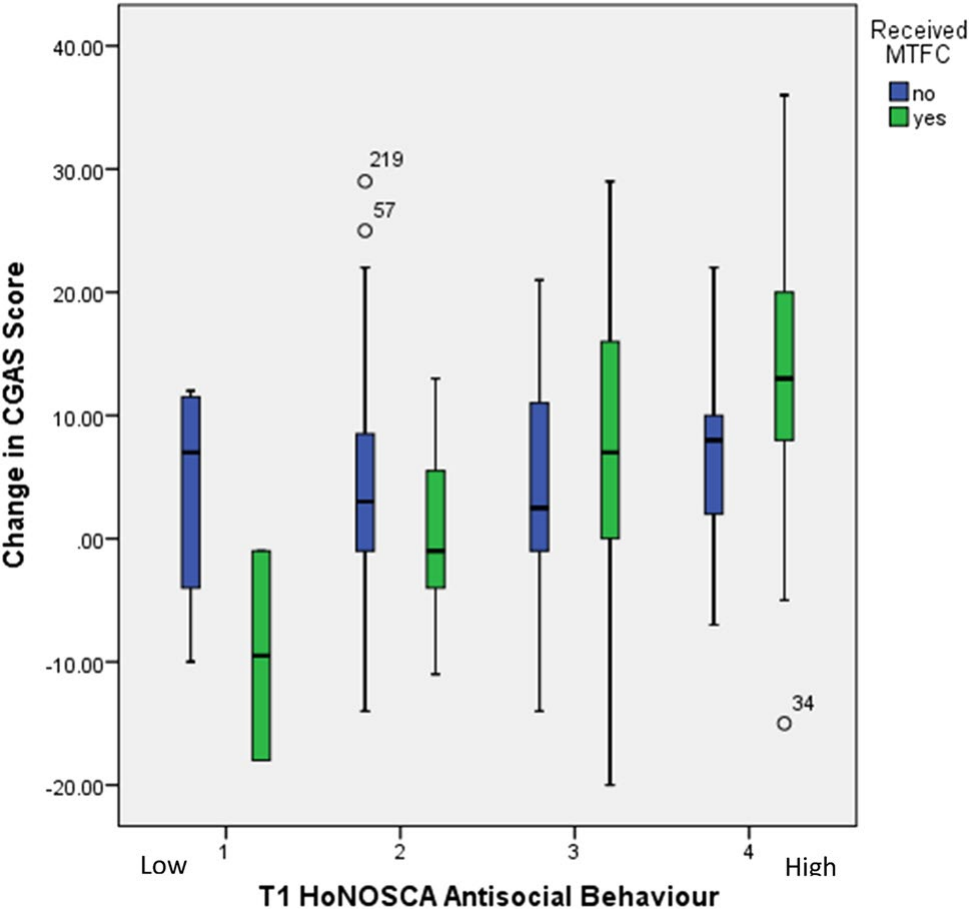
Change in CGAS score by initial antisocial rating

Figure 1 presents these results graphically comparing the average change in the CGAS score by the antisocial rating and type of intervention. In TAU the average change is positive and similar in all the ratings. In the MTFC group by contrast the change distributions for neighbouring ratings overlap but the average change rises steadily from negative to positive as the antisocial ratings increase from 1 (low antisocial behaviour) to 4 (high antisocial behaviour). We use the full antisocial rating to show that the trend is not dependent on the cut-off point chosen to define the antisocial group. The higher the initial rating the more the balance of advantage swung to MTFC.

### Can these results be explained by differences between those receiving MTFC and TAU?

Table 2 describes the distribution of variables which either distinguished most sharply between MTFC and TAU (notably age, being in residential care and t1 CGAS) or which together best predicted outcome (severity, t1 CGAS, offending, and mental health problems). Our key comparisons are between MTFC and TAU within the antisocial and less antisocial groups. Within these groups the only variables that we considered in this article, and which differed significantly between MTFC and TAU, were age at baseline (in both groups) and sex (in the low antisocial group only) and being initially in residential care (in the high antisocial group). None of these variables was significantly related to outcome.

**Table 2:** Selected variables at baseline by antisocial group and receipt of MTFC

	High antisocial group			Low antisocial group			Total		
	MTFC (*n* = 67)	TAU (*n* = 45)	p*	MTFC (*n* = 21)	TAU (*n* = 38)	p*	MTFC (*n* = 88)	TAU (*n* = 83)	p*
Mean age SD	12.70 1.63	13.62 1.50	0.003	12.14 1.77	13.68 1.77	0.002	12.57 1.67	13.65 1.62	0.000
Female	48 %	36 %	0.203	32 %	61 %	0.027	44 %	47 %	0.644
In Residential Care	45 %	64 %	0.041	38 %	61 %	0.099	43 %	63 %	0.011
Mean HoNOSCA SD	1.58 0.438	1.60 0.408	0.775	1.27 0.334	1.18 0.421	0.271	1.508 0.433	1.41 0.463	0.153
Mean CGAS SD	45.28 6.82	44.82 6.29	0.718	50.71 6.54	53.95 8.38	0.087	46.58 7.11	49.00 8.59	0.046
Offended	64 %	78 %	0.125	33 %	18 %	0.197	51 %	57 %	0.415
Mental health problems	12 %	18 %	0.358	18 %	10 %	0.338	14 %	14 %	0.876
Mean ‘Severity’ score SD	0.28 0.75	0.26 0.98	0.931	−0.14 1.15	−0.29 0.93	0.452	0.18 0.87	0.01 0.97	0.239

* Significance levels based on Chi square for percentages and t tests for means

In theory, differences in these distributions between MTFC and TAU could explain our results. To test this possibility we used propensity scoring based on logistic regressions using all the variables in Table 2 (and thus all the baseline variables used in this paper) and regression models using the last four variables (our covariates). The models and propensity scores were always based on the same variables but the weights given to these variables were calculated for the comparison being made (e.g. the propensity score used in comparing MTFC and TAU within the high antisocial group was based on a logistic regression run on the high antisocial group).

Table 3 gives the regression models for our four analytic groups with the last two columns giving separate models for MTFC and TAU. The latter suggest treatment interactions with MTFC changing the associations between outcomes on the one hand and mental health problems, prior offending, and the initial CGAS score on the other. (The apparent effect on the association with the initial CGAS is particularly striking and reflects a difference in the first order correlation between the t1 and t2 CGAS which is 0.56 in the TAU group to −0.004 in the MTFC one.). The inclusion of these interactions in a regression predicting the t2 CGAS for the whole sample raises the proportion of variance explained from 17 to 27 %.

**Table 3:** Regressions predicting CGAS outcome in different groups

	High antisocial group		Low antisocial group		Total sample	
	Beta		Beta		Beta	
	MTFC	TAU	MTFC	TAU	MTFC	TAU
Risk	−0.350**	−0.237	−0.286	−0.148	−0.254^*^	−0.170
Offending	−0.061	−0.353*	−0.123	−0.160	−0.052	−0.268**
Mental health problems	−0.443***	−0.011	−0.316	0.088	−0.337***	0.025
CGAS t1	−0.248*	0.401***	0.159	0.420*	−0.141	0.403***
Adjusted *R* ^2^	0.200***	0.311***	0.142	0.117	0.125**	0.331***

* ≤ 0.05, ** ≤ 0.01 *** ≤ 0.001

The equations in Table 3 allowed us to estimate separate ‘regression modelled effects’ (RMEs) for the antisocial and less antisocial groups. These were the expected difference in outcomes if all those in the relevant group had received MTFC as against all of them receiving TAU. Our calculations assumed that those whom we did not observe getting MTFC (or conversely TAU) would have responded to it according to their characteristics in the same way as those who did get it (i.e., the regression coefficients with the t2 CGAS would have been the same).

**Fig. 2:**
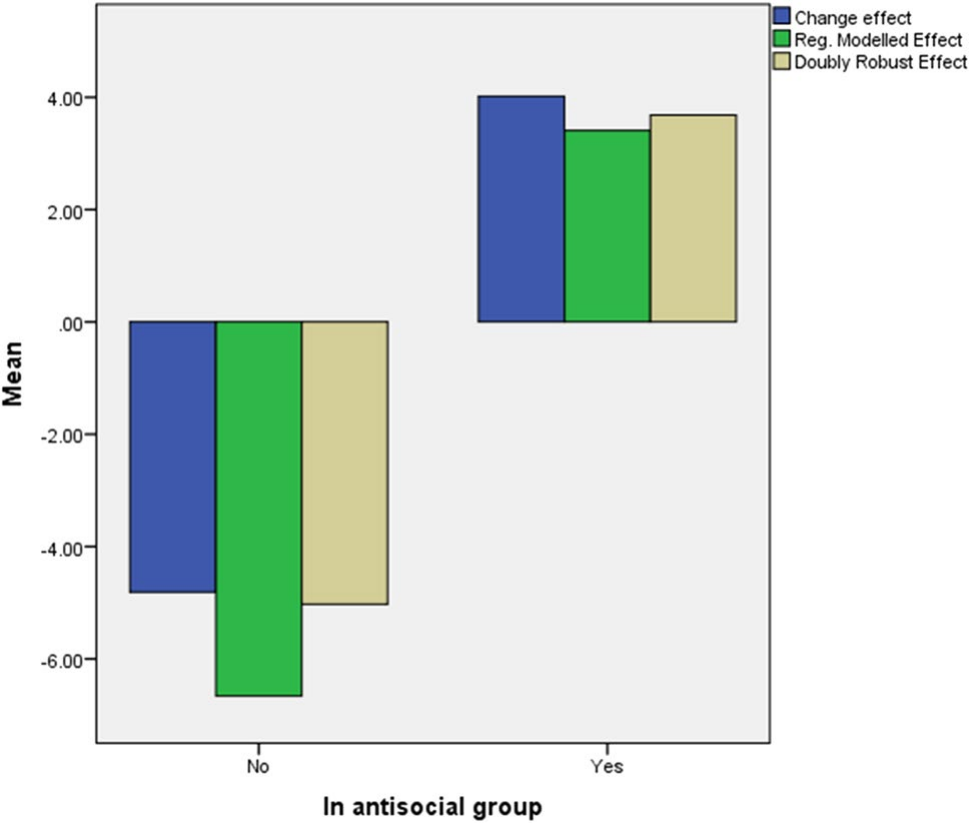
Estimated mean difference in effects of MTFC and TAU on CGAS by antisocial group

We then combined this modelling with inverse propensity score weighting. This allowed us to calculate a doubly robust estimate (DRE), which is correct if either the propensity or regression model is correct [23]. Figure 2 gives the modelled and doubly robust estimates for the antisocial and less antisocial groups. For comparison we give the unadjusted change effect (CE) (i.e., the average change observed among those receiving MTFC minus the average change observed among those receiving TAU).

Both the RME and DRE were positive and significant in the antisocial group (RME = 3.41 se = 0.76, *p* < 0.001, DRE = 3.68, se = 1.58, *p* < 0.05). The effects were larger, negative but non-significant in the low antisocial group (RME = −6.66, se = 4.12, *NS*. DRE = −4.62, se = 2.95, *NS*). Five inverse propensity weights were outliers being five or more standard deviations from the mean; dropping them from the analysis slightly increased the effect sizes but did not change the significance levels.

Our estimates of effect therefore support the hypotheses that MTFC will benefit the high antisocial group but not the less antisocial group, where the trend suggests a negative effect.

### Was the effect in the antisocial group explained by changes in antisocial behaviour?

Within the antisocial group the mean antisocial scores on the HoNOSCA disruptiveness scales of those receiving MTFC fell from the moderate to severe range to the mild to moderate one (3.37 at t1 to 2.34 at t2). The corresponding fall among those receiving TAU was within the moderate to severe range (3.44–3.00). Thus while there was virtually no difference in antisocial score by treatment at t1, the difference at t2 was significant (*t* = 2.586, *df* = 169, *p* = 0.002).

Change in antisocial behaviour in the antisocial group is also strongly correlated with the t2 CGAS score (*r* = 0.649, *p* < 0.001). We tested its role in mediating the effect of MTFC on outcome finding that it reduced the direct association from *β* = 0.218 to *β* = 0.035 while yielding a significant estimated indirect effect (effect = 3.76, se = 1.21, *p* < 0.01 as calculated through the MEDIATE SPSS sub-routine written by Hayes [26]).

By contrast the average change in the other scales of the HoNOSCA was similar for both groups and small, with the mean rating for these other scales falling from 1.40 to 1.15 for MTFC and 1.44 to 1.26 for TAU, a far from significant difference between the two groups.

It remained possible that this lack of average effect concealed both positive and negative impacts. In a final series of exploratory analyses we examined the association between receipt of MTFC and change on these 12 other HoNOSCA scales. Each regression included the initial score on the scale, the change at t2 and receipt of MTFC as a dummy variable. We then examined the effect of adding change in antisocial behaviour. We expected that this addition would reduce the apparent positive effect of MTFC on variables where change was mediated by change in antisocial behaviour while revealing negative effects ‘masked’ by improvements in antisocial behaviour.

We found an almost significant association between receipt of MTFC and improvement in relationship with carers (*β* −0.122, *p* = 0.065 in the trimmed sample and *β* = 0.167, *p* = 0.049 in the antisocial sub-sample) but this was greatly reduced when change in antisocial behaviour was added. By contrast there was a significant negative association (*β* = −0.193, *p* = 0.002 in trimmed sample but *β* −0.078, *p* = 0.32 in the antisocial sub-sample) between receipt of MTFC and improvement in emotional symptoms. However, this apparently negative effect of MTFC on emotional symptoms only became apparent when account was taken of change in antisocial behaviour.

These unpredicted findings are highly tentative because of the number of tests done but can provide hypotheses for further research. They suggest that where the young person displays antisocial behaviour, MTFC may improve relationships with carers because of its association with improved behaviour. By contrast improvements in behaviour brought about by MTFC may mask a negative effect of MTFC on emotional symptoms and this effect may be particularly apparent when there is no antisocial behaviour to improve. These results could help to explain why the more antisocial group did better if they received MTFC while the less antisocial group did worse.

Taken together these findings provide strong support for our hypothesis that MTFC will benefit antisocial young people through its effect on antisocial behaviour. Further exploratory analysis suggested that taking account of the positive effect of MTFC on antisocial behaviour masked its significant indirect positive effect on the HoNOSCA scale measuring relationships with carers (i.e., it had a positive effect on this through its impact on antisocial behaviour). It also masked a significant direct negative effect on the HoNOSCA scale measuring emotional symptoms (in other words it would have a negative effect on these but for its positive effect on antisocial behaviour). These unpredicted results are considered in our discussion below.

## Discussion

Previous trials of MTFC have focussed on young people with antisocial behaviour and reported positive effects on their behaviour [3, 4, 7] and on their birth mothers [8, 9]. The latter may change their perceptions of the young people and show improvements in their own well-being. These benefits seem limited to—or most pronounced among—those with the most challenging behaviour [11–13]—and are probably related to changes in an underlying variable of antisocial behaviour [24]. In two of the five studies they did not persist after the MTFC placement [8, 25].

In keeping with this evidence our study confirms our hypotheses that MTFC affects antisocial behaviour. More specifically, it suggests that the positive effects are limited to those displaying a high degree of antisocial behaviour; and that any impact on other symptoms is mediated by changes in this behaviour. It also suggested an unpredicted positive effect on relationships with carers. Its evidence on sustainability was inconclusive. Those who had left the placement were doing significantly worse at follow-up. This suggests a diminution of effect but our design prevented us from confirming it.

This evidence supports Miklowitz’s [27] suggestion that MTFC, like other similar interventions based on social learning, is a systemic intervention improving antisocial behaviour, and thus relationships with carers. There is thus a benign cycle with lowered stress leading to less difficult behaviour. Since the process depends on the reduction of difficult behaviour its effects should be restricted to those initially displaying it. Since it is systemic its maintenance will depend on the environment to which the young people move.

This benign circle is potentially very powerful. Some young people in the MTFC antisocial group displayed gains of 25 points or more on the CGAS—far greater than any found in the control group. Why then was the average adjusted advantage even to this group less than four points? One reason may be that we compared the short-term TAU placements with the long-term TAU ones. Given that MTFC is a systemic intervention its advantage may not be sustained if the follow-on placements are not superior to those found in TAU.

A second reason for the modest adjusted effect may be that Miklowitz’s ‘stress model’ should include the possibility that MTFC can itself be stressful. Some of the young people in our study resented MTFC which they saw as punitive or only suitable for younger children, while others resented the time-limits and the need to leave carers who were important to them. Even in the antisocial group four young people showed a drop of 15 points or more on the CGAS, a greater fall than any found in the TAU group. These ‘downsides’ may explain the worse outcomes of MTFC for those with mental health problems, along with the suggestion in our data that there is a direct negative effect on ‘emotional symptoms’ masked by a positive indirect effect through a reduction in antisocial behaviour.

These considerations would also explain the apparent conflict between our findings and reports of a positive effect of MTFC on psychotic symptoms [6] and depression [5]. These reports do not refer to a significant difference at follow-up but rather to the significantly different rate of change apparently produced by MTFC. They thus reflect the initial starting point as well as the end point. Despite randomisation the initial starting points for both depression and psychotic symptoms were higher in these analyses and in the case of psychotic symptoms significantly so. The findings may therefore reflect a transient negative impact on symptoms measured after the start of MTFC (as was always the case with the psychotic symptoms and half the time with the depressive ones) and a subsequent return to normal assisted by the impact on antisocial behaviour.

The general lesson is that to increase the effect size we must understand why some recipients of MTFC improve markedly and others do not or even get worse. If we do not do this, the results of future randomised trials will depend on the composition of the populations from which they are drawn, the length of follow-up and other unmeasured influences we do not understand. By contrast enhanced understanding should lead to better targeting and also, perhaps, to keeping some young people longer and providing others with more determined support on release. Our findings do not suggest that MTFC is an ineffective intervention. They do suggest that we do not at present know how to use it most effectively.

### Strengths and limitations

We have reported on a mixed methods study combining RCT and observational components with the latter containing initially unbalanced groups. This has complicated our analysis, but does not invalidate its conclusions, which are supported by our ‘doubly robust’ estimates. The pattern of change over time in the different groups fits previous evidence and bears out our hypotheses. If either of our propensity or our regression models is correct then the pattern can be interpreted in terms of cause and effect. The groups we compare are balanced on the baseline variables found to predict outcome. Theoretically there could be an unmeasured confounder which is strongly associated with both outcome and the nature of the intervention experienced, which would invalidate our inferences from the results. However, given the coherence of our evidence and the range of variables on which we collected data we think it extremely unlikely that such a confounder exists.

### Practical implications

Our findings add to the evidence that interventions based on social learning theory can reduce antisocial behaviour but that these benefits can be hard to maintain and limited in scope. They also suggest that children with low antisocial behaviours may do better with another approach. Thus, what may be needed is not a standard protocol exposing a wide variety of children to a broadly similar regime, but an approach which tailors the regime to a more theoretically informed assessment of each child or adapts it to their response. Until further evidence is forthcoming we recommend that

- MTFC is reserved for the antisocial young people who were its original target and benefit from it. It should not be given to those who are not antisocial
- Less intensive forms of the model (TFCO) which focus on training and supporting ‘ordinary’ foster carers [28] can continue to be tried, as they are less costly than MTFC, and avoid the possibly unsettling effects of changes of placement
- Further research should not simply establish whether or not an intervention works but should routinely examine for whom it does or does not work, what its negative effects may be, how and under what conditions it works and how long its effects last.
